# Gastro-intestinal symptoms as clinical manifestation of peritoneal and retroperitoneal spread of an invasive lobular breast cancer: report of a case and review of the literature

**DOI:** 10.1186/1471-2407-6-193

**Published:** 2006-07-19

**Authors:** G Franceschini, A Manno, A Mulè, A Verbo, G Rizzo, D Sermoneta, L Petito, P D'alba, C Maggiore, D Terribile, R Masetti, C Coco

**Affiliations:** 1 Dept of Surgery, Università Cattolica del Sacro Cuore, Rome, Italy; 2 Dept of Pathology, Università Cattolica del Sacro Cuore, Rome, Italy

## Abstract

**Background:**

Distant spread from breast cancer is commonly found in bones, lungs, liver and central nervous system. Metastatic involvement of peritoneum and retroperitoneum is unusual and unexpected.

**Case presentation:**

We report the case of a 67 year-old-woman who presented with gastrointestinal symptoms which revealed to be the clinical manifestations of peritoneal and retroperitoneal metastatic spread of an invasive lobular breast cancer diagnosed 15 years before.

**Conclusion:**

To the best of our knowledge, the case presented is the third one reported in literature showing a wide peritoneal and extraperitoneal diffusion of an invasive lobular breast cancer. The long and complex diagnostic work up which led us to the diagnosis is illustrated, with particular emphasis on the multidisciplinary approach, which is mandatory to obtain such a result in these cases. Awareness of such a condition by clinicians is mandatory in order to make an early diagnosis and start a prompt and correct therapeutic approach.

## Background

Invasive lobular breast cancer (ILC) takes origin in the milk-producing glands of the breast and is the most common histological breast cancer after the ductal carcinoma (DC), accounting for 8–14% of cases [1]. The typical histologic picture is characterized by small, regular, non-cohesive cells arranged in the so called "Indian file" appearance [2]; the neoplastic cells infiltrate the parenchyma around non-neoplastic ducts, inducing little connective tissue response [3]. Being physical examination and mammography often non-specific, contrast-enhanced magnetic resonance imaging (MRI) represents the gold standard for a correct diagnosis [4]. ILC has a higher tendency than DC to be multi-focal and bilateral [5]. Also the pattern of metastatic spread differs significantly between these 2 kinds of breast tumours, with a more common occurrence of unusual location of distant neoplastic foci, especially in the gastrointestinal tract, the genitourinary system and the peritoneum or retroperitoneum, secondary to ILC. This event is unexpected, usually with a long interval after the initial diagnosis of ILC, and the presenting symptoms as well as the endoscopic and the radiographic pictures are often non-specific. These conditions lead to a frequent delay in diagnosis which prevents the prompt starting of systemic treatment necessary to obtain a good control of symptoms. The case reported gives an example of this unusual metastatic diffusion and of the complex diagnostic work up which led us to he diagnosis.

## Case presentation

**Figure 1:**
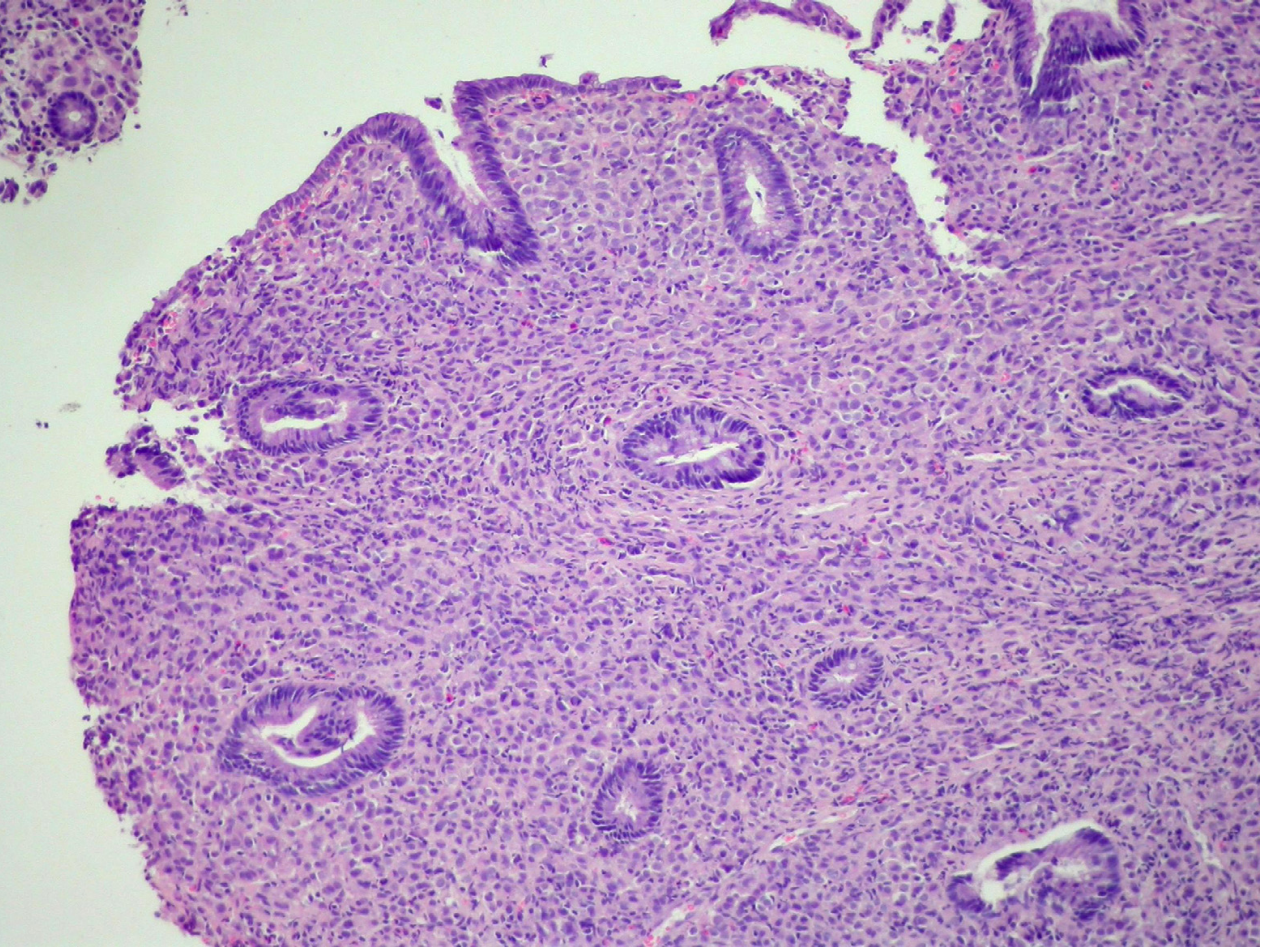
Rectum biopsy: Infiltration by ILC with focal targettoid arrangement around rectal glands. The rectal glandular epithelium shows no dysplastic changes (original magnification ×100).

**Figure 2:**
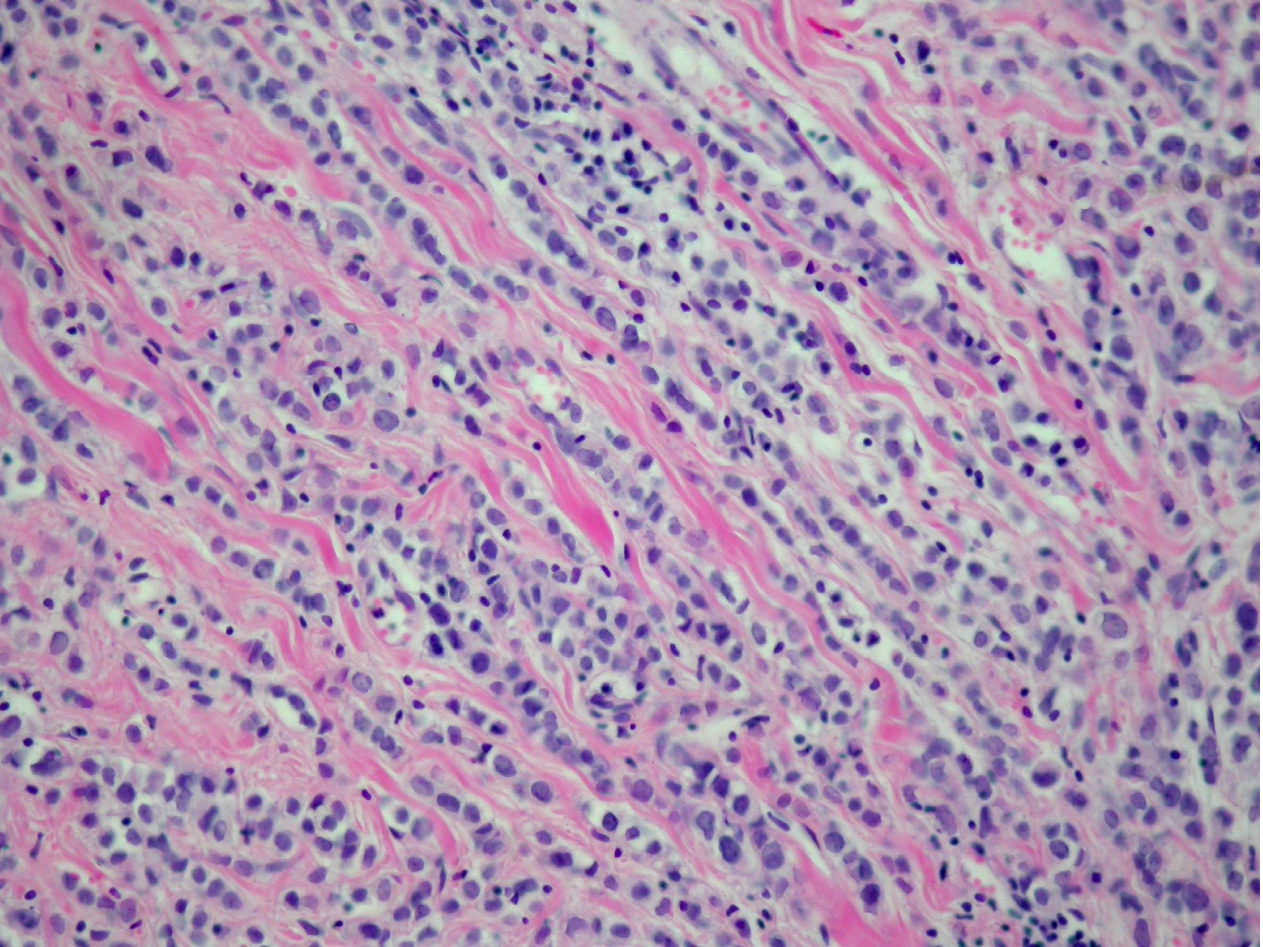
A detail of the typical "Indian file" growth pattern of breast lobular invasive carcinoma (original magnification ×200).

**Figure 3:**
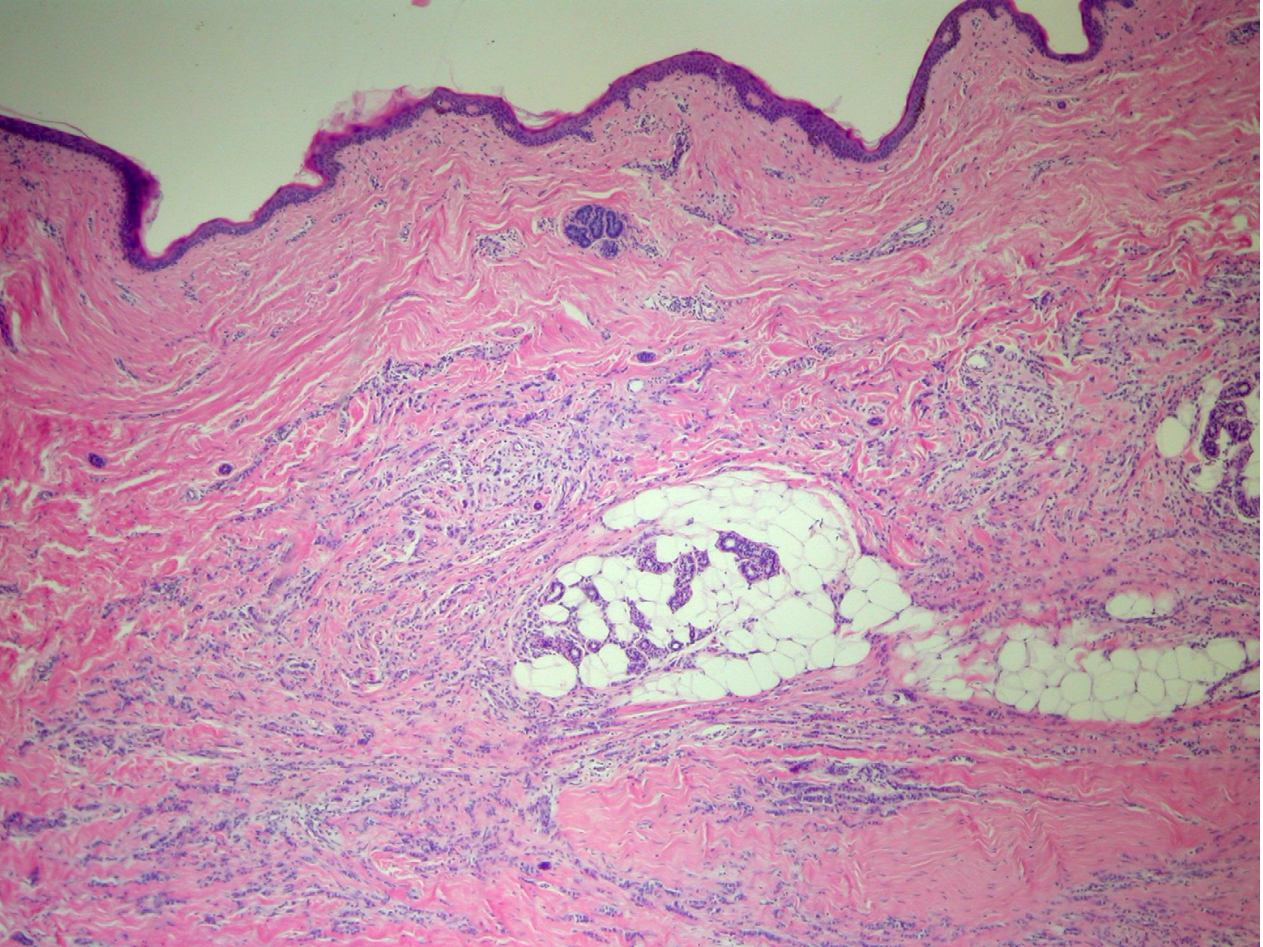
Vaginal biopsy: Infiltration by ILC with typical "Indian file" growth pattern. (original magnification ×100).

**Figure 4:**
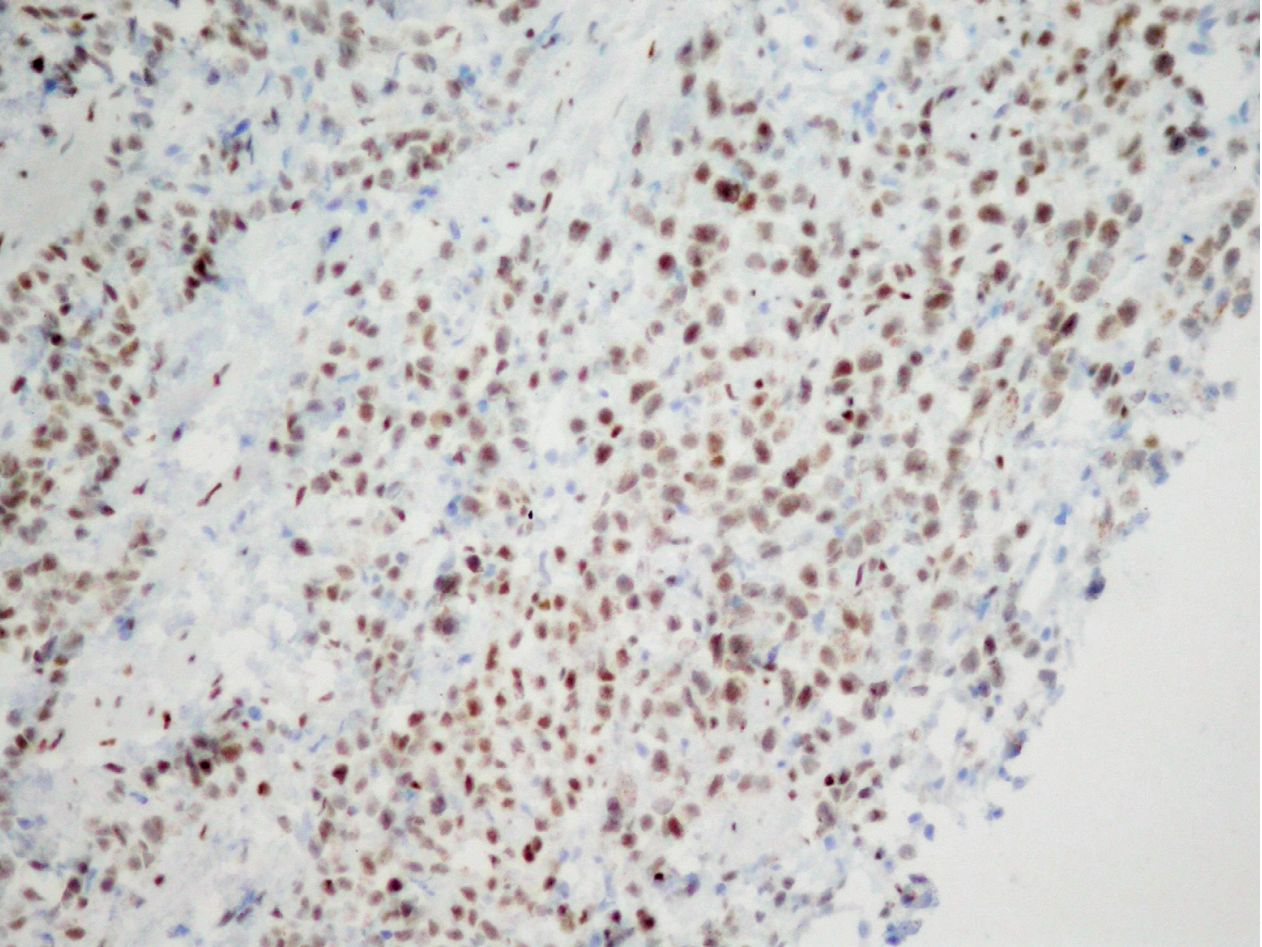
Positive ER staining confirming the breast origin of the metastasis. (original magnification ×200).

A 67-year-old woman underwent right modified radical mastectomy and axillary lymph node dissection for carcinoma of the breast 15 years ago. Histological examination of the tumour revealed a 4 cm invasive lobular carcinoma of histological grade 2. Two of the 20 lymph nodes examined were infiltrated by tumour cells. Immunohistochemistry for oestrogen and progesterone receptors showed weak staining of 20% of cancer cells for both receptors. There was no evidence of distant metastases at the time of diagnosis. The patient received six cycles of adjuvant chemotherapy (cyclophosphamide 500 mg/m^2^, mitoxantrone 10 mg/m^2^, 5-fluorouracil 500 mg/m^2^, every 21 days) and was on tamoxifen. Ten years later a local recurrence occurred, and the patient underwent partial resection of the thorax wall followed by reconstruction by transverse rectus abdominis musculocutaneus (TRAM) flap technique. No adjuvant treatment was given. The patient came to our attention complaining of a 4-month history of diffuse abdominal pain associated to constipation, tenesmus and sporadic rectal bleeding. On physical examination, the patient was pale but moderately nourished. The mastectomy bed, the controlateral breast, and both axilla were normal. Abdominal examination showed no palpable mass or ascites. At digital examination the rectum appeared stenotic from about 6 cm above the anal verge, but without evidence of endoluminal masses. Haematological analysis and biochemical parameters including liver and renal function tests were within the normal range. The urine cytology revealed micro-hematuria. The patient was submitted to a rectosigmoidoscopy which showed a diffuse thickening of the anterior wall of the rectum, which determined mild stenosis beginning 7 cm above the anal verge, without evidence of endoluminal masses. The mucosa which lined the anterior rectal wall was hyperaemic and easily bleeding. The posterior wall of the vagina showed diffuse thickening at vaginal endoscopy. The histological examination of multiple biopsies taken during rectosigmoidoscopy, revealed an extensive infiltration by scarcely cohesive neoplastic cells with "Indian file" features and focal targettoid arrangement around rectal glands (Fig 1, 2). The vaginal biopsy confirmed a prevalent "Indian file" neoplastic growth pattern (Fig. 3). In both biopsies malignant cells were small, with atypical nuclei and vacuolated cytoplasm, often with "signet ring" morphology. The rectal glands and the vaginal epithelium showed no atypias A panel of selected immunohistochemical markers was used to confirm the metastatic nature of the neoplastic mass and its site of origin. Immunohistochemical stainings for oestrogen and progesterone receptors (Fig 4), GFCDP-15, C-ERB-B2 and CK 7 were positive. Otherwise, they resulted negative for CK 20, WT-1, CA-125 and CDX-2. This immunohistochemical pattern, together with the typical morphological picture showed above, let us confirm the diagnosis of metastatic location from ILC.

A computerised tomography (CT) scan of the abdomen and pelvis showed how the pelvic cavity was almost completely occupied by neoplastic tissue which infiltrated the rectal wall, causing marked stenosis, both the ovaries, the fundus of the vagina and the left lateral wall of the bladder, with involvement of the left ureter and concurrent hydronephrosis. Multiple enlarged lymph-nodes were identified in the perirectal fat, along the common iliac artery and the obturator chain and in the inter-aortocaval space. The liver and the chest appeared normal. Mammographic and ultrasonographic picture of the left breast and of both axilla were normal. On the basis of the diagnosis of wide peritoneal and extraperitoneal metastatic spread of ILC, the patient entered a protocol of systemic chemotherapy and hormonal therapy after the placement of a J ureteral stent to pass through the stenosis evidenced by the CT scan and confirmed by cystoscopy.

## Discsussion

It is well known that the pattern of metastatic spread differs dramatically between ductal and lobular breast cancer [6,7]. In particular, the retrospective series by Borst et al, comparing metastatic rates between these 2 histological subtypes in 2605 cases of breast cancer, showed statistically significant differences for metastases in the gastrointestinal tract (4,5% in ILC vs 0,2% in DC), gynaecological organs (4,5% in ILC vs 0,8 in DC) and peritoneum/retroperitoneum (3,1% in ILC vs 0,6% in DC) [7]. The 50% of metastasis to the gastrointestinal tract from ILC affects the small bowel [7] while involvement of the large bowel, especially of the rectum, is infrequent [8] and often multifocal [9]. The metastatic spread may develop into the peritoneum, in the form of small or confluent nodules [10], or into the retroperitoneum, often resulting in ureteric obstruction and hydronephrosis [11]. Only 19 cases of bladder location of metastases from breast cancer are reported in the English literature till 2000 and only 33% were secondary to ILC among them [12]. The gynaecological organs are not spared by metastatic spread from ILC, expecially the uterus and the ovary, which account for the 80% of all gynaecological metastastases from ILC [13]. Concomitant multiple peritoneal and extraperitoneal locations of metastatic disease from ILC, as in the case reported, is an even more rare evenience; to the best of our knowledge, apart from the present report, only other two cases have been published in literature till know [11,14].

The most representative series of metastatic ILC, focused on the specific sites of involvement, are summarized in table I [see additional file 1].

The clinical manifestations in such cases are usually non-specific, and strikingly similar to that of primary gastrointestinal and genitourinary malignancies [15,16]. The median interval between presentation of metastasis and diagnosis of ILC is five to six years [8,15]; an interval of more than 10 years, up to 15 years as in our case, have rarely been described [8,15,17]. Both the above reported conditions make differential diagnosis between a primary tumour and metastatic ILC, difficult. Moreover, if the clinical picture is often inconclusive, also conventional imaging techniques and endoscopy show potential limitations in obtaining a differential diagnosis [8,15,16,18,19]. At CT scan our case showed a picture that may be considered typical, made of huge parietal thickening of several pelvic organs such as rectum, bladder and vagina, as well as bilateral hydronephrosis due to retroperitoneal involvement. This characteristic infiltrative pattern of diffusion is non-specific for metastatic desease from breast cancer, as may resemble the picture of primary neoplasms of gastrointestinal or genitourinary systems. A recent report failed to demonstrate any diagnostic advantage of MRI and PET [11]. Endoscopy with biopsy appears an essential step in the diagnostic work up, as it is showed in the present case: we found the typical endoscopic picture of a colorectal metastatic lesion, characterized by diffuse thickening and rigidity of the rectal wall, but no endoluminal masses. Histological finding on biopsy samples gave us the definitive confirmation of the metastatic nature of the lesion. As already stressed in most recent reports, the common finding of a "signet ring" pattern neoplasia can be misleading, as it may suggest a primitive origin from the gastro-intestinal tract [20], but the lack of dysplasia or atypia of the rectal glandular epithelium let us state the metastatic nature of the malignancy, and the specific origin from ILC was strongly suggested by the typical "Indian file" arrangement of the neoplastic cells [19,21]. A further confirmation came from immunohistochemistry. Metastatic breast carcinomas are often positive for Cytokeratin 7(CK7), GCDFP-15, ER and/or PgR. This was true in our case. Moreover, the immunohistochemical negativity for CA125 and WT1 ruled out a possible ovarian origin and the negativity for Cytokeratin 20(CK20) and CDX2 excluded the colic one [22,23]. (Table 2) [see additional file 1]

The therapeutic approach to these patients is still object of debate. Surgery is mainly indicated to solve stenotic or hemorrhagic evolution of the colorectal locations. Although some authors suggest a potential benefit of surgical debulking procedures followed by chemotherapy [24], systemic treatment (chemotherapy, endocrine treatment or both) is considered the therapy of choice in patients with metastases to the peritoneum and retroperitoneum involving multiple organs [8,15]. The prognosis remains poor with a partial response in about 50% of patients and a median survival time from diagnosis of about 1 year [8]. Many authors suggest the importance of early diagnosis, which enables prompt initiation of systemic treatment, with or without surgery, thus giving advantage in terms of survival.

## Conclusion

The case reported is peculiar, as it focuses on an extremely rare condition which, if ignored, leads to a delayed treatment, thus worsening the prognosis in terms of survival. The long and complex diagnostic workup described, draws the attention to the difficulty of making a correct diagnosis, also because the long interval after the initial diagnosis of breast cancer makes the occurrence of metastasic disease an unexpected event.

This report stresses the importance for the clinician to be aware of the eventuality of a wide peritoneal/extraperitoneal metastatic diffusion, when dealing with a patient with previous diagnosis of ILC and presenting for gastrointestinal or genitourinary symptoms. Only bearing in mind such a possibility, the aggressive, multidisciplinary approach necessary to make diagnosis can be early undertaken and an optimal therapeutic approach promptly performed.

## Competing interests

The author(s) declare that they have no competing interests.

## Authors' contributions

Franceschini G Concept and design

Manno A Concept and design

Mulè A Histology and immunoassays

Maggiore C Histology and immunoassays

Verbo A Acquisition, analysis and interpretation of data

Rizzo G Acquisition, analysis and interpretation of data

Sermoneta D Drafting of the manuscript

Petito L Critical revision

D'alba P Critical revision

Masetti R Final revision and approval

Coco C Final revision and approval

All authors read and approved the final manuscript.

## Pre-publication history

The pre-publication history for this paper can be accessed here:


